# Avemar and *Echinacea* extracts enhance mobilization and homing of CD34^+^ stem cells in rats with acute myocardial infarction

**DOI:** 10.1186/s13287-015-0171-5

**Published:** 2015-09-14

**Authors:** Maha Abdelmonem, Samar H. Kassem, Hala Gabr, Amira A. Shaheen, Tarek Aboushousha

**Affiliations:** Biochemistry Department, Faculty of Pharmacy, Cairo University, Cairo, Egypt; Biochemistry Department, Faculty of Physical Therapy, October 6 University, Cairo, Egypt; Clinical Pathology Department, Faculty of Medicine, Cairo University, Cairo, Egypt; Pathology Department, Theodor Bilharz Research Institute, Cairo, Egypt

## Abstract

**Introduction:**

Activation of endogenous stem cell mobilization can contribute to myocardial regeneration after ischemic injury. This study aimed to evaluate the possible role of Avemar or *Echinacea* extracts in inducing mobilization and homing of CD34^+^ stem cells in relation to the inflammatory and hematopoietic cytokines in rats suffering from acute myocardial infarction (AMI).

**Methods:**

AMI was developed by two consecutive subcutaneous injections of isoprenaline (85 mg/kg). AMI rats were either post-treated or pre- and post-treated daily with oral doses of Avemar (121 mg/kg) or *Echinacea* (130 mg/kg). In whole blood, the number of CD34^+^ cells was measured by flow cytometry and their homing to the myocardium was immunohistochemically assessed. Serum creatine kinase, vascular endothelial growth factor, interleukin-8 and granulocyte macrophage colony stimulating factor were determined on days 1, 7 and 14 after AMI. Sections of the myocardium were histopathologically assessed.

**Results:**

Rats pre- and post-treated with Avemar or *Echinacea* exhibited substantial increases in the number of circulating CD34^+^ cells, peaking on the first day after AMI to approximately 13-fold and 15-fold, respectively, with a decline in their level on day 7 followed by a significant increase on day 14 compared to their corresponding AMI levels. Only post-treatment with *Echinacea* caused a time-dependent increase in circulating CD34^+^ cells on days 7 and 14. Such increases in circulating CD34^+^ cells were accompanied by increased homing to myocardial tissue 14 days after AMI. Interestingly, pre- and post-treatment with Avemar or *Echinacea* substantially increased serum creatine kinase on day 1, normalized its activity on day 7 and, on continued treatment, only *Echinacea* markedly increased its activity on day 14 compared to the corresponding AMI values. Moreover, both treatments modified differently the elevated serum vascular endothelial growth factor and the lowered granulocyte macrophage colony stimulating factor levels of the AMI group but did not affect the level of interleukin-8. These results were supported histopathologically by reduced inflammatory reactions and enhanced neovascularization.

**Conclusion:**

Avemar and *Echinacea* extracts can effectively induce mobilization and homing of CD34^+^ stem cells to the myocardial tissue and thus may help in stem cell-based regeneration of the infarcted myocardium.

## Introduction

Myocardial infarction (MI) is one of the major causes of cardiovascular morbidity and mortality. MI results in loss of cardiomyocytes, scar formation, ventricular remodeling and eventually heart failure. Although current pharmacological and surgical interventions have led to improved survival of patients, they fail to regenerate dead myocardium and/or prevent deterioration of cardiac function [1]. In last decade, stem cell (SC) therapy has emerged as a potential new strategy for incurable and life-threating MI. The ultimate goals of SC therapy are myocardial regeneration and neovascularization leading to clinical improvement without severe adverse effects. Mechanisms involved in the endogenous SC-associated myocardial regeneration include the mobilization of SCs from the bone marrow (BM) and other putative “niches” (such as skeletal and cardiac muscles), cytokine-guided homing with subsequent engraftment into the ischemic area, and finally the transdifferentiation into functional cardiomyocytes. These tissue-committed SCs circulate in peripheral blood at low number and can be mobilized by ischemia-related inflammatory and hematopoietic cytokines, such as granulocyte colony-stimulating factor (G-CSF), granulocyte macrophage colony-stimulating factor (GM-CSF), interleukin (IL)-8, vascular endothelial growth factor (VEGF), and stromal cell-derived factor-1 (SDF-1) [2–5]. The levels of these cytokines were found to be significantly higher in patients with acute myocardial infarction (AMI) and were correlated positively with the number of circulating CD34^+^ SCs [4]. However, such endogenous responses unfortunately do not offer a sufficient regenerative solution of damaged myocardium. Therefore, the need for SC therapy is a must. Basically, the efficacy of SC therapy in regenerative medicine depends on sufficient recruitment of available cells (either exogenously administered populations or endogenously mobilized residents) to the target tissue.

Although SC transplantation is the most common means to replenish impoverished SC pools, their applications are restricted by the limited availability of SC sources, the excessive cost and the anticipated difficulties of clinical translation and regulatory approval. Thus, regenerative therapy should not be limited to this approach but should instead seek for a strategy that retrieves the initial healing capacity of a tissue [2]. In this regard, pharmacological activation of endogenous SCs already present in a patient’s body from either the blood or a tissue-specific niche and their homing into the injury sites is a promising approach for therapeutic success. This technique has the potential to provide new therapeutic options for in situ tissue regeneration. Such options would be less costly and complex than approaches requiring ex vivo cell manipulation [2, 3]. In this context, using medicinal plant products for activation of endogenous SCs represents an emerging field of regenerative medicine in health and disease.

In the current study, two natural products, namely Avemar and *Echinacea*, were selected to investigate their possible role in supporting SC biology. Avemar is a product of industrial fermentation of wheat germ with a standardized content of benzoquinone and plant flavonoids that has been reported as safe and effective anticancer and immunomodulatory adjuvant therapy for human cancer [6]. Avemar also has a potential role to attenuate the cardiovascular symptoms induced by hypertension or diabetes. The proposed mechanisms were attributed to its actions in inhibition of cyclooxygenase isoforms and upregulation of endogenous antioxidants [7]. On the other side, *Echinacea purpurea* is one of the *Echinacea* species which has been widely used for its anti-inflammatory and antioxidant activity in addition to a profound immunostimulatory action on a number of human immune cells, such as macrophages and peripheral blood mononuclear cells [8]. Clinical trials confirmed the efficiency of *Echinacea purpurea* extracts in inflammatory diseases, including those of an infectious nature [9]. *Echinacea* has been shown to improve the regeneration process of damaged tissues where it can intensify the immunological angiogenesis which could be of high benefit in wound healing and in future cardiology as a preventive for MI [10]. Thus, we hypothesized that treatment with Avemar and *Echinacea* may help in regeneration of damaged myocardium via stimulating SCs. In this regard, the effect of these natural products on activation of endogenous SCs cannot be separated from their already known antioxidant, anti-inflammatory and immunomodulatory activities, as all of these activities are speculated to synergistically drive tissue repair and regeneration.

Thus, this study was directed for the first time to investigate the possible effect of Avemar or *Echinacea* on enhancing CD34^+^ SC mobilization, and homing in relation to inflammatory and hematopoietic cytokines such as VEGF, IL-8 and GM-CSF in a rat model of AMI.

## Methods

### Animals

Male Wistar rats, weighing 170 ± 20 g, were obtained from animal facility of the Faculty of Pharmacy, Cairo University. The animals were housed in plastic cages under controlled temperature (25 ± 2 °C) and a constant 12-h light/12-h dark cycle throughout the experiment. A standard rat chow diet and water were allowed ad libitum. The investigation complies with the Guide for Care and Use of laboratory Animals published by the US National Institutes of Health (NIH publication No. 85–23, revised 1996) and was approved by the Ethics Committee for Animal Experimentation at the Faculty of Pharmacy, Cairo University.

### Materials

Isoprenaline was purchased from Sigma-Aldrich Chemical Co. (USA) and freshly prepared before injection in normal saline at a concentration of 100 mg/ml. Avemar (standardized fermented wheat germ extract) was purchased from American Biosciences Inc. (USA) and was freshly prepared in cold distilled water at a concentration of 500 mg/100 ml. *Echinacea purpurea* extract was kindly provided by Mepaco-Medifood Co. (Egypt) and was freshly prepared in distilled water at a concentration of 500 mg/100 ml.

### Experimental design

Rats were randomly divided into six groups. Group I (n = 6) received subcutaneous injections of saline and served as normal control. Group II (n = 18) received subcutaneous injection of isoprenaline (85 mg/kg) for 2 consecutive days at 24-h time intervals for the development of infarct-like myocardial lesions [11]. Group III (n = 12) and V (n = 12) received isoprenaline as in group II followed by post-treatment daily with oral doses of Avemar (121 mg/kg) [12] or *Echinacea* (130 mg/kg) [13], respectively, for 14 days. Group IV ( n = 18) and VI ( n = 18) were pretreated daily with an oral dose of Avemar (121 mg/kg) or *Echinacea* (130 mg/kg), respectively, for 14 days prior to the induction of AMI and treatment continued for another 14 days after the development of AMI.

At the end of treatment, animals were anesthetized with intraperitoneal injection of thiopental (50 mg/kg). Blood was collected from the retro-orbital sinus at the following intervals: in the normal group, 1 day after the last injection; in groups II (AMI group), IV and VI (pre- and post-treated groups), on days 1, 7 and 14 after induction of myocardial infarction (six animals per time); in groups III and V (post-treated groups), on days 7 and 14 after induction of myocardial infarction (six animals per time).

The collected blood was divided into two halves. One half was used for the separation of serum where aliquots of the separated serum were stored at −20 °C for subsequent determination of creatine kinase (CK), IL-8, GM-CSF and VEGF. The other half was collected in EDTA-coated tubes for determination of CD34^+^ cell counts by flow cytometry. After blood collection, the animals were euthanized and the hearts were rapidly excised, washed with cold saline and kept in formalin for immunohistochemical and histopathological investigations.

### Flow cytomytric analysis of CD34^+^ cells

Flow cytometric analysis of CD34^+^ cells was performed at different time intervals immediately after blood collection. Monoclonal CD34 antibody (10 μl) conjugated to fluorescein isothiocyanate (Miltenyi Biotec Inc., USA) was added to 100 μl EDTA blood. The suspension was incubated for 30 minutes in the dark in the refrigerator (2–8 °C). VersaLyse lysing solution (1 ml; Immunotech SAS, France) was added and vortex was carried out immediately for 1 second. The suspension was incubated for 10 minutes at room temperature protected from light. Data were acquired using a Beckman Coulter (Epics XL, Switzerland) flow cytometer. The results are expressed as a percentage of positive events (CD34^+^ cells) in relation to all events acquired by gating (10,000 events).

### Determination of serum CK activity

Serum CK activity was determined according to the manufacturer’s instructions given with the CK kit (Stanbio Laboratory, USA). This method optimizes the reaction by reactivation of CK activity with N-acetyl-L-cysteine. The data was expressed as IU/l.

### Determination of serum VEGF, IL-8 and GM-CSF

Serum levels of VEGF, IL-8 and GM-CSF were determined by a quantitative sandwich enzyme-linked immunosorbent assay (ELISA) technique according to the manufacturer’s instructions. Kits for VEGF and GM-CSF were obtained from R&D Systems (USA) while that for IL-8 was obtained from Glory Science Co. (USA). The concentration of samples was determined from the standard curves and the results were expressed as pg/ml.

### Histological examination

The rat hearts of different groups were isolated 1 day and 14 days after AMI, washed with ice-cold saline and fixed in 10 % formol saline for 24 hours. Specimens were processed for paraffin embedding and 4 μm sections were prepared. The sections were stained with a hematoxylin and eosin (H&E) stain and were examined by the light electric microscope. Histological changes were evaluated by a pathologist unaware of the type of treatment. The severity and extent of MI were observed for each case. The findings were classified into: nil, mild (focal myocyte damage or small multifocal degeneration with slight degree of inflammation), moderate (extensive myofibrillar degeneration and diffused inflammatory reaction), or severe (necrosis with diffused inflammatory reaction) [14].

### Measurement of capillary density

The effect of Avemar and *Echinacea* on neovascularization was evaluated by counting the number of capillary vessels within myocardial sections with H&E staining under light microscopy. A capillary vessel was defined as a vessel with a diameter less than 200 μm. The number of capillaries was counted under microscopy (magnification ×100) for five random fields in myocardial sections and presented as mean of blood vessels per unit area (0.2 mm^2^). The capillary count of these sections was compared with the AMI group [15].

### Immunohistochemical investigation of CD34^+^ cells

Immunohistochemical analysis for CD34^+^ cells was performed 14 days after AMI development on formalin-fixed, paraffin-embedded cardiac tissue sections using the NovoLink Polymer Detection System (Leica Microsystems, UK) [16]. Endogenous peroxidase activity was neutralized using the Peroxidase Block. This was followed by application of the Protein Block to reduce nonspecific binding of the primary antibody and the polymer. The sections were subsequently incubated with diluted rat CD34 antibody (US Biological Co., USA). Post Primary Block was used to enhance the penetration of the subsequent polymer (IgG-Poly-HRP). Peroxidase activity was developed with 3,3-diaminobenzidine working solution which produces brown precipitate at the antigen site. Slides were counterstained with hematoxylin. Examination was performed using Image Analyzer (Olympus, USA) where CD34^+^ cells in the myocardial tissue were counted in ten fields per slide and the average number of CD34^+^ cells was calculated.

### Immunohistochemical investigation of α-SMA

Immunohistochemical analysis for alpha-smooth muscle actin (α-SMA)-positive cells was performed 14 days after AMI development on formalin-fixed, paraffin-embedded cardiac tissue sections using a DAKO immunostainer (DAKO, USA) according to the manufacturer’s protocol. Sections (4 μm) were deparaffinized and rehydrated through graded alcohols. Endogenous peroxidase activity was blocked with 0.3 % hydrogen peroxide for 10 minutes, and antigens were retrieved for 20 minutes in 10 mmol/l citrate buffer, pH 6.0, at 98 °C. Nonspecific binding was blocked with 10 % normal goat serum for 20 minutes. The tissue slides were incubated with the antibody (1:500 dilution; DAKO) for 65 minutes at 25 °C. Then the slides were incubated with a ChemMate DAKO Envision system (K5007) for 30 minutes at 25 °C and subsequently reacted with 3,3′-diaminobenzidine as a chromogen substrate. The nucleus was counterstained using Mayer hematoxylin. A score from 0 to 3+ was applied; where 0 = absence of α-SMA positive cells in 5 fields, 1 = 1–5 cells/5 fields, 2 = 6–10 cells/5 fields and 3 = more than 10 cells/5 fields (magnification ×100).

### Statistical analysis

Data are expressed as mean ± standard error. The significance of differences between groups was determined using one-way analysis of variance followed by a post-hoc procedure (Tukey’s Test). Correlation between variables was determined using Pearsons correlations. Statistical analysis was performed using SPSS version 17 software (SPSS®, USA). For all statistical tests, a value of *P* < 0.05 was considered statistically significant.

## Results

### Flow cytometric analysis of CD34^+^ cells

**Fig. 1:**
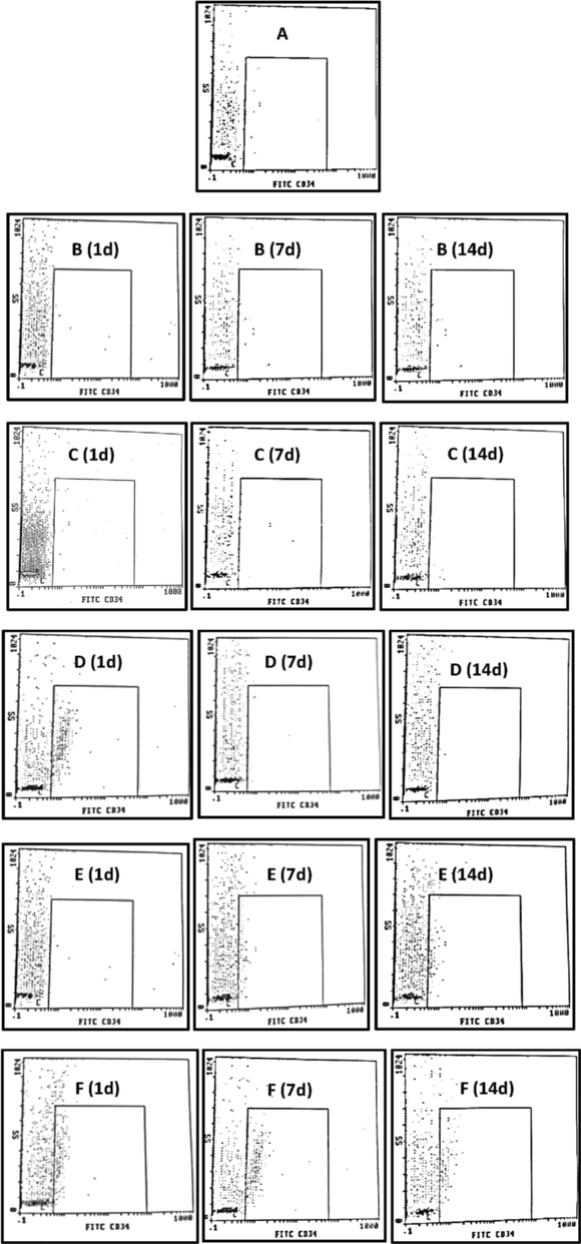
Flow cytometric plots of peripheral CD34^+^ cell counts at different time intervals from AMI. **a** Normal; **b** AMI group; **c** AMI post-treated with Avemar; **d** AMI pre- and post-treated with Avemar; **e** AMI post-treated with *Echinacea*; **f** AMI pre- and post-treated with *Echinacea*. *d* Day after AMI

Data given in Table 1 and Fig. 1 show the time course of circulating CD34^+^ cells. In the AMI group, these cells increased on day 7 and peaked on day 14 from the MI development to fivefold the normal value (*P* < 0.001). Pre- and post-treatment with Avemar or *Echinacea* resulted in a substantial increase in the circulating CD34^+^ cells in peripheral blood peaking on day 1 after AMI to about 13- and-15 fold (*P* < 0. 001), respectively, then with a decline on day 7 followed by significant increase on day 14 compared to their corresponding AMI values. However, post-treatment with Avemar failed to affect the circulating level of these cells compared to the corresponding AMI group. On the other hand, post-treatment with *Echinacea* was more effective; it progressively increased the circulating level of CD34^+^ cells by 2.3-fold on day 7 followed by 3.2-fold (*P* < 0.001) on day 14 compared to the corresponding AMI group.

**Table 1:** Effect of Avemar or *Echinacea* treatment on circulating CD34^+^ cells at different time intervals from AMI development (n = 6/group)

	Day 1	Day 7	Day 14
Normal		0.28 ± 0.023	
AMI	0.28 ± 0.028	0.91 ± 0.068	1.59 ± 0.14^a^
AMI post-treated with Avemar	0.28 ± 0.028	0.78 ± 0.07	1.4 ± 0.08^a^
AMI pre- and post-treated with Avemar	3.65 ± 0.275^ab^	1.38 ± 0.095^a^	3.17 ± 0.196^ab^
AMI post-treated with *Echinacea*	0.28 ± 0.028	2.14 ± 0.09^ab^	5.1 ± 0.196^ab^
AMI pre- and post-treated with *Echinacea*	4.22 ± 0.35^ab^	1.34 ± 0.099^a^	3.65 ± 0.099^ab^

Data are expressed as mean ± standard error. ^a^Significantly different from the normal group at *P* < 0.01. ^b^Significantly different from the corresponding AMI value at *P* < 0.01. *AMI* acute myocardial infarction

### Serum CK activity

**Fig. 2:**
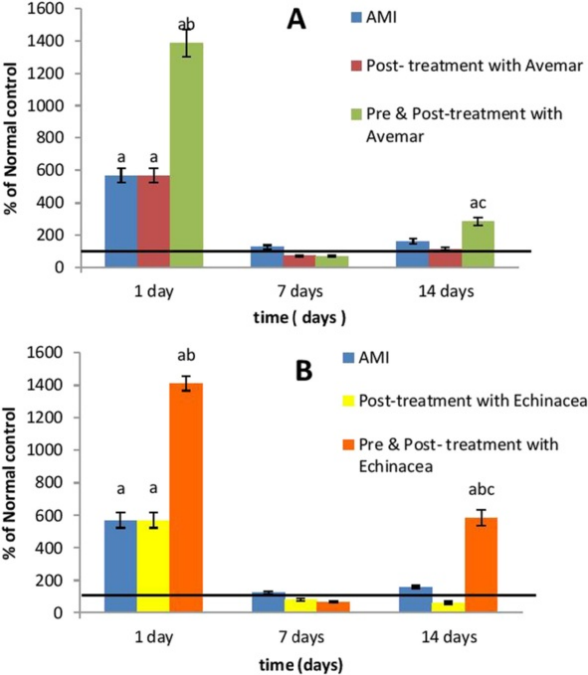
Effect of (**a**) Avemar treatment or (**b**) *Echinacea* treatment on serum CK activity in rats with acute myocardial infarction (AMI) at different time intervals from the infarction development. Values are expressed as a percentage of normal. ^a^ Significant difference from the normal group at *P* < 0.05. ^b^ Significant difference from the corresponding AMI group at *P* < 0.05. ^c^ Significant difference from the corresponding post-treated group at *P* < 0.05

Induction of AMI resulted in a fivefold increase (*P* < 0.001) of serum CK activity 1 day after infarction development when compared to the normal group. This activity was then comparable to the normal group on days 7 and 14. Post-treatment of AMI rats with Avemar or *Echinacea* had no effect on serum CK activity when compared to their corresponding AMI values. On the other hand, animals pre- and post-treated with Avemar or *Echinacea* showed substantial increases in serum CK on day 1 after infarction development reaching 243 % and 247 % (*P* < 0.001), respectively, of the infarction values. On continued treatment, the serum CK activities returned to normal levels after 7 days then increased again insignificantly with Avemar and significantly with *Echinacea* (364 % at *P* < 0.001) on day 14 after AMI development relative to their corresponding infarction values, as shown in Table 2 and Fig. 2.

**Table 2:** Effect of Avemar or *Echinacea* treatment on serum creatine kinase activity (IU/l) at different time intervals from AMI development (n = 6/group)

	Day 1	Day 7	Day 14
Normal		329 ± 32	
AMI	1875 ± 154^a^	409 ± 45	527 ± 55
AMI post-treated with Avemar	1875 ± 154^a^	235 ± 19	371 ± 33
AMI pre- and post-treated with Avemar	4566 ± 274^ab^	219 ± 10	935 ± 83
AMI post-treated with *Echinacea*	1875 ± 154^a^	273 ± 25	200 ± 25
AMI pre- and post-treated with *Echinacea*	4636 ± 144^ab^	216 ± 16	1922 ± 166^ab^

Data are expressed as mean ± standard error. ^a^Significantly different from the normal group at *P* < 0.01. ^b^Significantly different from the corresponding AMI value at *P* < 0.01. *AMI* acute myocardial infarction

### Serum VEGF

**Fig. 3:**
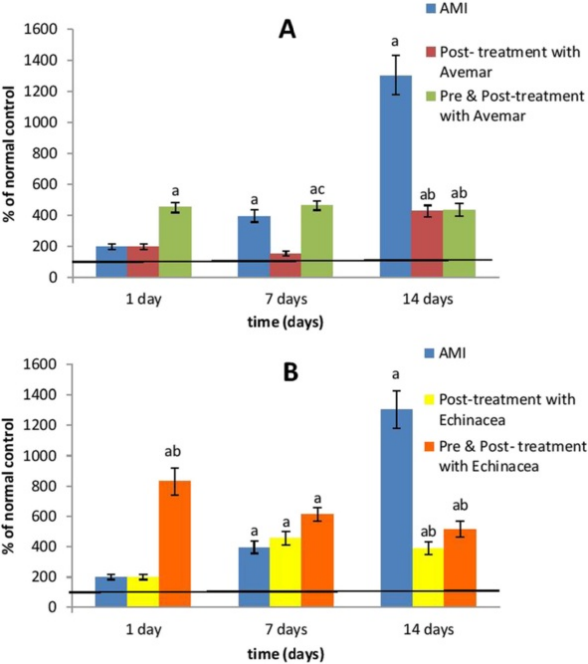
Effect of (**a**) Avemar treatment or (**b**) *Echinacea* treatment on the serum VEGF level in rats with acute myocardial infarction (AMI) at different time intervals from the infarction development. Values are expressed as a percentage of normal. ^a^ Significant difference from the normal group at *P* < 0.05. ^b^ Significant difference from the corresponding AMI control group at *P* < 0.05. ^c^ Significant difference from the corresponding post-treated group at *P* < 0.05

Data in Table 3 and Fig. 3 show that rats suffering from AMI had a progressive increase in VEGF level, reaching 13-fold (*P* < 0.001) the normal value on day 14 after infarction development. When AMI rats were post-treated with Avemar or *Echinacea*, serum VEGF decreased to one-third the corresponding AMI values (*P* < 0.001) on day 14 after infarction development. In groups pre- and post-treated with Avemar or *Echinacea*, the VEGF level increased by 4.5-fold (*P* < 0.001) and by 8.3-fold (*P* < 0.001) the normal level, respectively, on day 1 after infarction development. Moreover, on day 14 these treatments decreased serum VEGF levels to 33 % and 39 % (*P* < 0.001), respectively, of their corresponding AMI values.

**Table 3:** Effect of Avemar or *Echinacea* treatment on serum vascular endothelial growth factor level (pg/ml) at different time intervals from AMI development (n = 6/group)

	Day 1	Day 7	Day 14
Normal		26.8 ± 2.1	
AMI	53.4 ± 4.7	106.3 ± 10.8^a^	349.7 ± 33.5^a^
AMI post-treated with Avemar	53.4 ± 4.7	41.4 ± 4	114.9 ± 9.8^ab^
AMI pre- and post-treated with Avemar	121.3 ± 8.8^a^	124.3 ± 7.5^a^	116.9 ± 11.1^ab^
AMI post-treated with *Echinacea*	53.4 ± 4.7	122 ± 12.3^a^	104 ± 10.9^ab^
AMI pre- and post-treated with *Echinacea*	222.5 ± 23.6^ab^	163.9 ± 12.2^a^	137.5 ± 14.2^ab^

Data are expressed as mean ± standard error. ^a^Significantly different from the normal group at *P* < 0.05. ^b^Significantly different from the corresponding AMI value at *P* < 0.05. *AMI* acute myocardial infarction

### Serum IL-8

**Fig. 4:**
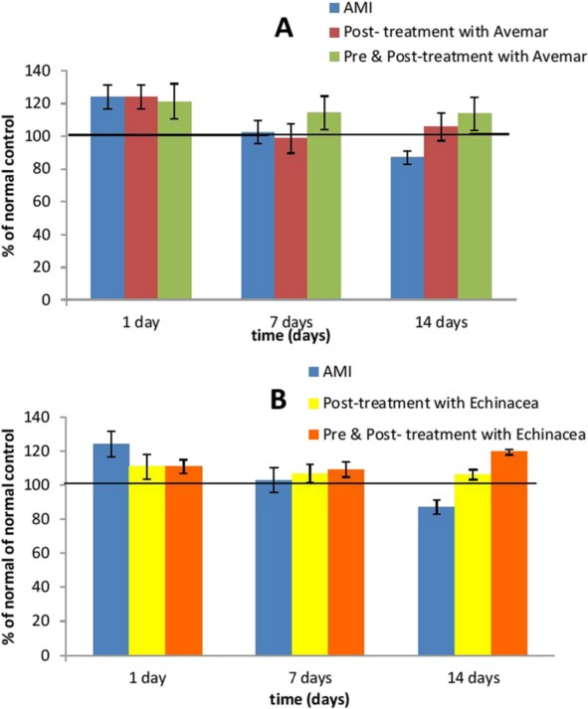
Effect of (**a**) Avemar treatment or (**b**) *Echinacea* treatment on the serum IL-8 level in rats with acute myocardial infarction (AMI) at different time intervals from the infarction development. Values are expressed as a percentage of normal

Induction of AMI with isoprenaline resulted in an insignificant increase of the serum IL-8 level on day 1 after AMI development in comparison with the normal group. Moreover, neither Avemar nor *Echinacea* treatment could alter the serum IL-8 level significantly compared to their corresponding AMI group (Table 4 and Fig. 4).

**Table 4:** Effect of Avemar or *Echinacea* treatment on serum interleukin-8 level (pg/ml) at different time intervals from AMI development (n = 6/group)

	Day 1	Day 7	Day 14
Normal		361 ± 11.7	
AMI	448 ± 26.5	371 ± 25.8	314 ± 14.5
AMI post-treated with Avemar	448 ± 26.5	356 ± 31.8	382 ± 31.3
AMI pre- and post-treated with Avemar	438 ± 38.7	413 ± 36.5	411 ± 36.8
AMI post-treated with *Echinacea*	448 ± 26.5	385 ± 20.2	383 ± 10.4
AMI pre- and post-treated with *Echinacea*	400 ± 14.8	394 ± 15.9	431 ± 5.5

Data are expressed as mean ± standard error, with no significant difference between groups. *AMI* acute myocardial infarction

### Serum GM-CSF

**Fig. 5:**
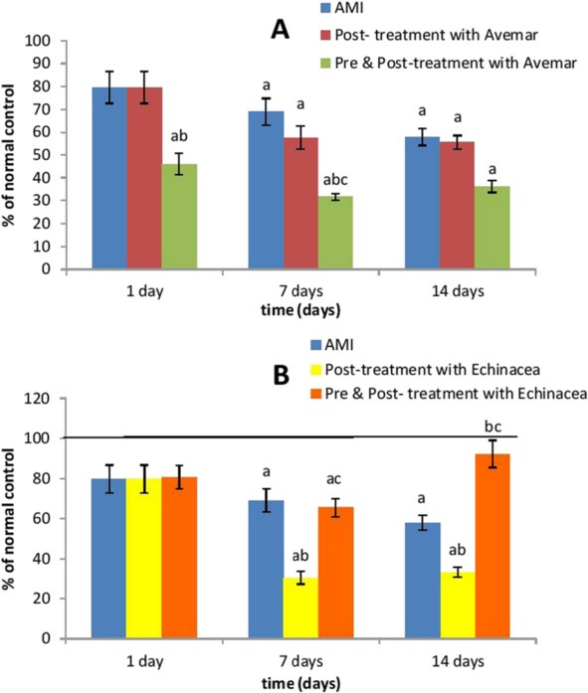
Effect of (**a**) Avemar treatment or (**b**) *Echinacea* treatment on serum GM-CSF in rats with acure myocardial infarction (AMI) at different time intervals from the infarction development. Values are expressed as a percentage of normal. ^a^ Significant difference from the normal group at *P* < 0.05. ^b^ Significant difference from the corresponding AMI control group at *P* < 0.05. ^c^ Significant difference from the corresponding post-treated group at *P* < 0.05

Induction of AMI in rats resulted in a progressive decrease in serum GM-CSF compared to the normal level throughout the time course of investigation. Post-treatment with Avemar did not affect the decrease in the level of GM-CSF caused by AMI. On the other hand, post-treatment with *Echinacea* resulted in more decreases in the serum GM-CSF levels on days 7 and 14 than their infarction values, reaching 30 % (*P* < 0.001) of the normal value. Pre- and post-treatment with Avemar resulted in more pronounced decreases in serum GM-CSF to 46 %, 32 % and 36 % (*P* < 0.001), on days 1, 7 and 14 after AMI development, respectively, compared to the normal group. However, pre- and post-treatment with *Echinacea* did not affect the serum GM-CSF level on days 1 and 7, but significantly increased its level to 159 % (*P* < 0.001) on day 14 compared to their corresponding AMI values (Table 5 and Fig. 5).

**Table 5:** Effect of Avemar or *Echinacea* treatment on the serum granulocyte macrophage colony-stimulating factor level (pg/ml) at different time intervals from AMI development (n = 6/group)

	Day 1	Day 7	Day 14
Normal		23.45 ± 1.97	
AMI	18.68 ± 1.63	16.2 ± 1.37^a^	13.58 ± 0.87^a^
AMI post-treated with Avemar	18.68 ± 1.63	13.5 ± 1.2^a^	13.02 ± 3.66^a^
AMI pre- and post-treated with Avemar	10.8 ± 1.06^ab^	7.4 ± 0.35^ab^	8.48 ± 0.58^a^
AMI post-treated with *Echinacea*	18.68 ± 1.63	7.13 ± 0.78^ab^	7.77 ± 0.59^ab^
AMI pre- and post-treated with *Echinacea*	18.89 ± 1.36	15.38 ± 1.07^a^	21.66 ± 1.6^b^

Data are expressed as mean ± standard error. ^a^Significantly different from the normal group at *P* < 0.05. ^b^Significantly different from the corresponding AMI value at *P* < 0.05. *AMI* acute myocardial infarction

### Correlation between circulating CD34^+^ cells and serum CK activity and cytokines

**Fig. 6:**
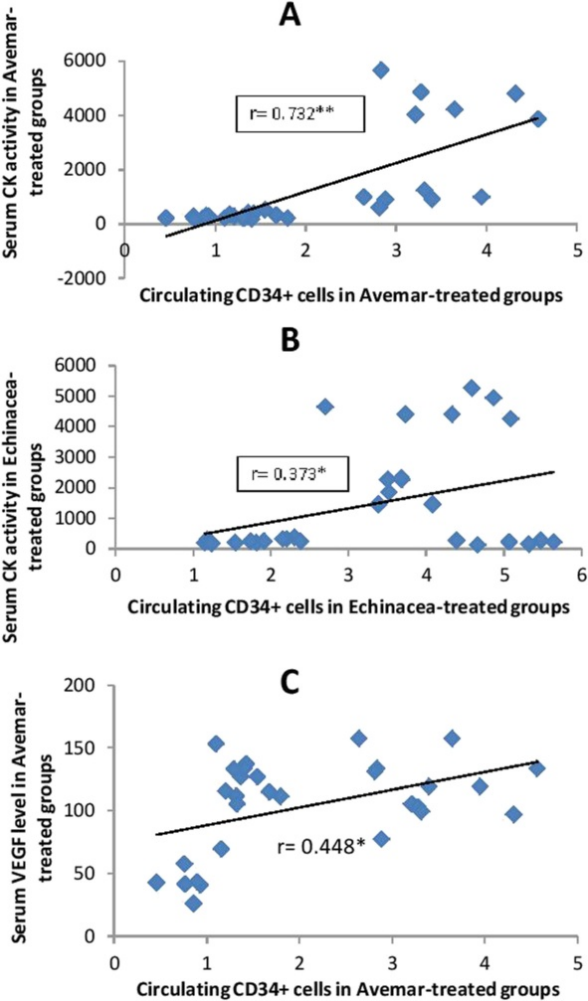
Correlation between circulating CD34^+^ cells and serum CK activity or serum VEGF level. **a** Correlation between circulating CD34^+^ cells and serum creatine kinase (CK) activity in Avemar-treated groups. **b** Correlation between circulating CD34^+^ cells and serum CK activity in *Echinacea*-treated groups. **c** Correlation between circulating CD34^+^ cells and serum vascular endothelial growth factor (VEGF) in Avemar-treated groups. Values of *P* < 0.05 are considered statistically significant: * significant at *P* < 0.05. ** significant at *P* < 0.01

When the potential relationship between circulating CD34^+^ cells and serum CK activity was examined in the experimental groups (Fig. 6a and b), simple regression analysis revealed a positive correlation between CD34^+^ counts and serum CK activity in groups treated with Avemar or *Echinacea* (r = 0.732 and 0.373, respectively; *P* < 0.05). Moreover, a positive correlation was also found between the serum level of VEGF and circulating CD34^+^ in Avemar-treated groups (r = 0.448; *P* < 0.05; Fig. 6c). No significant relationship was found between these cells and other cytokines in the Avemar- or *Echinacea*-treated groups.

### Immunohistochemical investigation of CD34^+^ cells

**Fig. 7:**
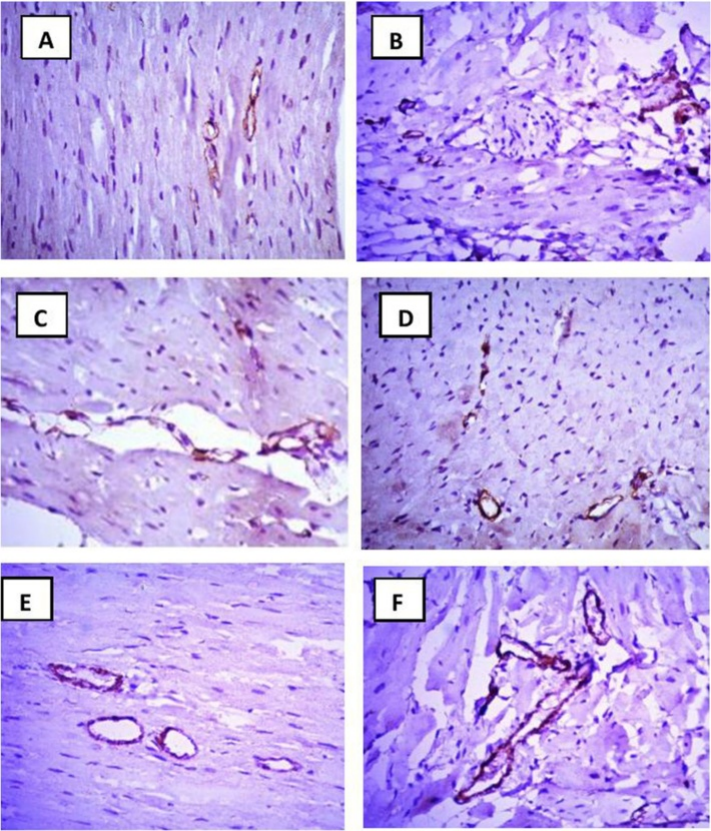
Immunohistochemical analysis for CD 34^+^ cells 14 days after infarction development. **a** Normal; **b** AMI group; **c** AMI post-treated with Avemar; **d** AMI pre- and post-treated with Avemar; **e** AMI post-treated with *Echinacea*; **f** AMI pre- and post-treated with *Echinacea*. Magnification ×400

**Fig. 8:**
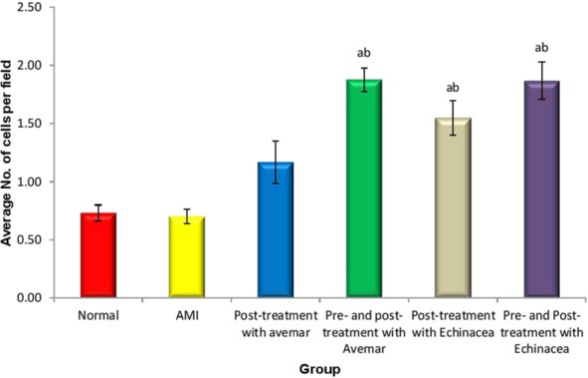
Average numbers of CD34^+^ cells in myocardial tissue 14 days after acute myocardial infarction (AMI) development. ^a^ Significant difference from the normal group at *P* < 0.05. ^b^ Significant difference from the AMI group at *P* < 0.05

The AMI group did not show any significant change in the number of CD34^+^ cells in myocardial tissue on day 14 when compared to the normal group (Figs. 7 and 8). Post-treatment with Avemar resulted in a slight increase in the number of CD34^+^ cells in myocardial tissue. On the other hand, pre- and post-treatment with Avemar caused a greater significant increase in CD34^+^ cell homing by 268 % (*P* < 0.001) on day 14 after infarction development than in the AMI group. Post-treatment with *Echinacea* increased CD34^+^ cell homing to 221 % (*P* < 0.001) and its pre- and post-treatment resulted in more homing at the myocardium reaching to267% (*P* < 0.001) of their AMI group.

### Immunohistochemical investigation of α-SMA-positive cells

**Fig. 9:**
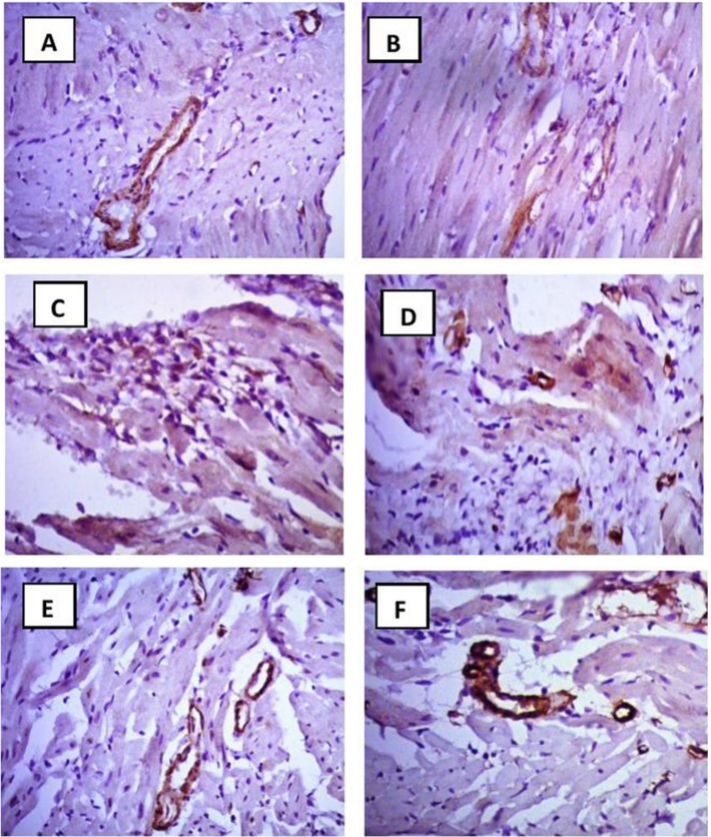
Immunohistochemical investigation of α-SMA positive cells 14 days after infarction development. **a** Normal; **b** AMI group; **c** AMI post-treated with Avemar; **d** AMI pre- and post-treated with Avemar; **e** AMI post-treated with *Echinacea*; **f** AMI pre- and post-treated with *Echinacea*. Magnification ×400

Positive staining for α-SMA was present only in the wall of coronary vessels of the control group. In the AMI group, few cells showing positive staining for α-SMA were recognized. On the other hand, homing of CD34^+^ cells at the myocardium in response to treatment was accompanied by higher expression of α-SMA 14 days after infarction. α-SMA-positive cells were increased in Avemar-treated groups with both vascular and separate scattered myofibroblast-like cells. In *Echinacea* pre- and post-treated rats, especially after 14 days of infarction, α-SMA-positive cells mostly gained histological features characteristic of pericytes in the wall of newly formed capillaries (Table 6 and Fig. 9).

**Table 6:** Effect of Avemar or *Echinacea* treatment on α-SMA-positive cells 14 days after infarction development

	Score of α-SMA-positive cells
Post-treatment with Avemar	+3
Pre- and post-treatment with avemar	+2
Post-treatment with *Echinacea*	+2
Pre- and post-treatment with *Echinacea*	+1

A score from 0 to 3+ was applied, where 0 = absence of alpha-smooth muscle actin (α-SMA)-positive cells in 5 fields, 1 = 1–5 cells/5 fields, 2 = 6–10 cells/5 fields and 3 = more than 10 cells/5 fields

### Histopathology

**Fig. 10:**
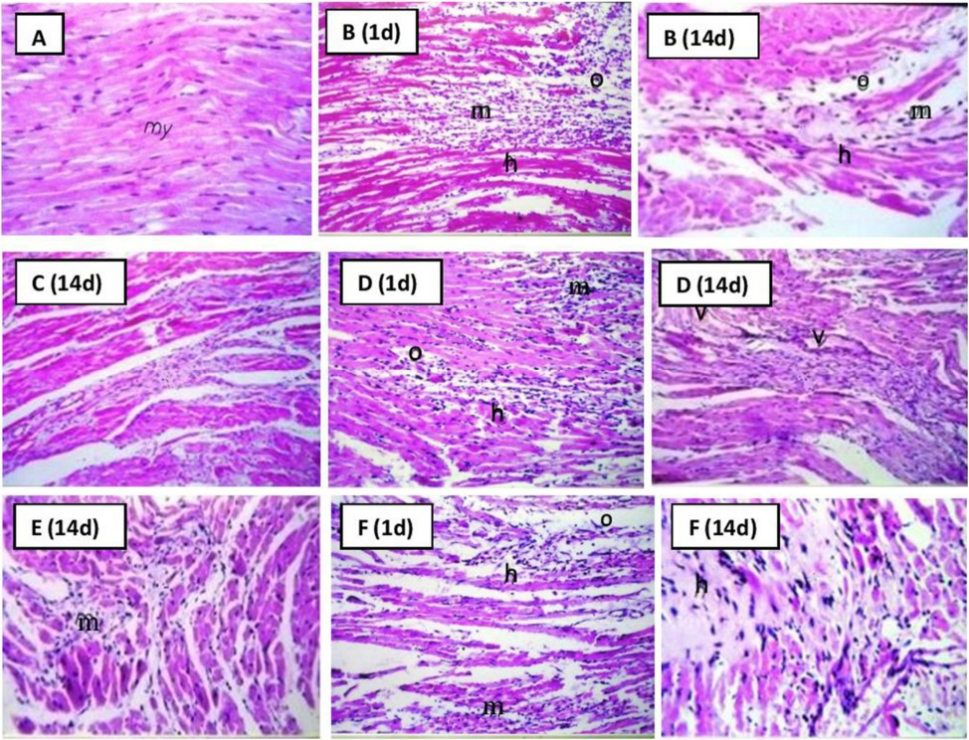
Histopathological analysis of myocardial tissue after AMI development (stained by H&E stain). **a** Normal; **b** AMI group; **c** AMI post-treated with Avemar; **d** AMI pre- and post-treated with Avemar; **e** AMI post-treated with *Echinacea*; **f** AMI pre- and post-treated with *Echinacea*. *d* Days after AMI; *h* hyalinization, *m* inflammatory cells infiltration, *o* edema, *v* newly formed blood capillaries

Histopathological analysis of the AMI group revealed a severe inflammatory reaction on day 1 after infarction development. This was represented by cardiac muscular degeneration, inflammatory cell infiltration, edema and hyalinization. Fourteen days after infarction, the inflammatory reaction was moderate and the natural healing process had started. Treatment with Avemar after infarction development resulted in a better healing of the myocardial tissue. In rats pre- and post-treated with Avemar, there was still a severe inflammatory reaction on day 1 after induction of AMI which disappeared after 14 days. The inflammation also disappeared in the group post-treated with *Echinacea* 14 days after AMI. In rats pre- and post-treated with *Echinacea*, there was a mild inflammatory reaction on days 1 and 14 after infarction development (Fig. 10).

### Capillary density

**Fig. 11:**
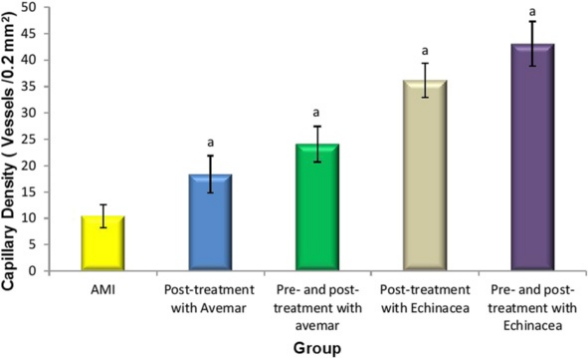
Effect of Avemar and *Echinacea* treatments on capillary density in the myocardium 14 days after acute myocardial infarction (AMI) development (mean ± standard error). ^a^ Significant difference from AMI group at *P* < 0.05

Regarding capillary density as index of neovascularization, H&E staining showed marked augmentation of neovascularization in Avemar- and *Echinacea*-treated groups. Semiquantitative analysis showed significant increases in newly formed capillaries in treated groups compared to the AMI group 14 days after infarction, with more remarkable values detected in the *Echinacea* post-treated and pre- and post-treated groups compared to other groups (Fig. 11).

## Discussion

In MI, stimulation of endogenous SC potential helps in healing and regeneration of damaged myocardial tissue [3]. A tissue regeneration approach that relies on endogenous SC mobilization and homing would be less costly and less complex than approaches that require substantial ex-vivo cell manipulation and use of artificial vehicles for cell delivery [2]. Dietary strategies for supporting SC biology represent an emerging field of nutritional medicine. The current study is the first one that investigates the possible beneficial role of two natural products, namely Avemar and *Echinacea*, on SC mobilization and homing, as well as their modulatory role on damaged myocardial tissue in relation to cytokines.

In this study, induction of AMI using isoprenaline resulted in increased circulating CD34^+^ cells with a peak on day 14 from infarction development. This finding is supported by previous studies in both animal models [17] and AMI patients [18]. The major problem of these mobilized CD34^+^ cells which limits their ability to regenerate the damaged myocardial tissue is their relatively low count in the circulation. Therefore, there is a great need for the discovery of new agents that induce mobilization of more SCs from their resident niches into the systemic circulation.

The data from the current study revealed that treatment with Avemar or *Echinacea* pre- and post-infarction efficiently induced great mobilization of CD34^+^ cells from their niches on day 1 and 14 after AMI. On the other hand, animals that received Avemar only after infarction development exhibited no change in the mobilization of SCs, while those receiving *Echinacea* showed more circulating CD34^+^ cells on days 7 and 14 compared to their corresponding AMI values. These results revealed the importance of Avemar as a prophylactic agent and *Echinacea* as both a prophylactic and therapeutic agent triggering an efficient mobilization of CD34^+^ cells which may, in turn, contribute to regeneration of damaged myocardium. Increased SC mobilization could be explained according to the established concept that, within the BM, SCs are anchored to stromal cells via integrin binding to cell adhesion molecules and via interaction with cell surface- or matrix-bound cytokines [19]. Disruption of this interaction represents the principal underlying mechanism of several currently applied mobilization strategies including colony-stimulating factors such as G-CSF and GM-CSF. Another underlying mechanism depends on increasing serum levels of growth factors which provide a gradient thus guiding SCs out into the blood, such as VEGF, erythropoietin, and IL-8 [3, 20]. The circulating levels of SCs in case of pre- and post-treatment with Avemar or *Echinacea* decreased to a certain extent after 7 days and then increased significantly again after 14 days, suggesting a migration of CD34^+^ cells to the infarcted myocardium.

Induction of AMI resulted in increased serum CK activity on day 1 after infarction suggesting a loss of cellular membrane integrity with myocardial cell damage [21, 22]. Importantly, our study demonstrated that intake of *Echinacea* or Avemar for 14 days before the development of MI significantly increased CK activity on day 1 after MI when compared to the untreated group. This increase may be explained in the light of SC-based cardiogenesis. SC differentiation is an energy-dependent process which requires constant metabolic signaling between the cytosol and nucleus to control gene expression. In particular, SC cardiogenesis involves robust electrical and functional activities that require additional energy requirements and coordinated signal communication [23]. CK isoforms are the major phosphotransfer circuit in adult cardiomyocytes which connects mitochondrial energetics with ATP-consuming processes in the nucleus and myofibrils [24]. Thus, cardiomyocyte differentiation is accompanied by enhancement of CK circuits and total activity to meet increased demands for energetic communication. In harmony with this theory, Chung and colleagues previously reported that treatment of differentiating SCs with bone morphogenetic protein 2, a cardiogenic growth factor, enhanced CK activity [24]. In support of this, a significant positive correlation between the circulating CD34^+^ cells and serum CK activity was observed in groups treated with Avemar or *Echinacea*.

Generally speaking, SC mobilization from the BM and other tissue niches into the circulation is guided by the interplay of several molecular and cellular mechanisms [3]. In the present study, a significant increase in serum VEGF was observed on day 7 after MI. In agreement with this result, a high serum VEGF level was previously found in clinical [25] and experimental [26] AMI. The elevated VEGF level at the acute phase of MI may be triggered through the activation of hypoxia-inducible factor-1 and/or it may be an adaptive mechanism for inducing neovascularization in ischemic myocardium [27]. In groups pre- and post-treated with Avemar or *Echinacea*, higher increases in serum VEGF were shown compared to the untreated group on day 1 after infarction development. Such increases might be responsible for the elevation in circulating CD34^+^ cells. Previous studies pointed to a positive correlation between plasma VEGF and the number of CD34^+^ cells in MI patients [28, 29]. Additionally, VEGF may also enhance the adhesive potential of progenitor cells within the myocardium, their homing capacity and subsequently their potential regenerative ability [29]. In spite of these previously mentioned beneficial roles, there is evidence that elevated levels of endogenous VEGF are associated with an inflammatory reaction in patients with acute coronary syndrome [27]. Likewise, atorvastatin treatment after MI resulted in a decline in the level of VEGF which was correlated with improvement of post-MI ventricular dysfunction and may account for the clinical benefit in the chronic phase of MI [27]. In the current study, the early increases in the VEGF level in groups pre- and post-treated with Avemar or *Echinacea* were followed by significant decreases on day 14; such effects may represent additional benefits of Avemar or *Echinacea* for AMI patients.

With regard to IL-8, our results showed insignificant increases of serum IL-8 level on day 1 after the last isoprenaline dose. Schömig and colleagues previously demonstrated an elevation of plasma IL-8 level in patients with AMI together with enhanced CD133 progenitor cell mobilization [30]. However, in the current study, the insignificant increases in plasma IL-8 could be attributed to the relatively short half-life of IL-8 (8 minutes in nonhuman primates) [31]. Moreover, neither Avemar nor *Echinacea* treatment could change serum IL-8 levels when compared to the AMI group. On the contrary, Kapai and colleagues reported that *Echinacea purpurea* preparations have a stimulatory effect on the production of IL-8 by mononuclear leukocytes in vitro [9]. Additionally, Ernesto and colleagues found substantial differences in the gene expression of IL-8 according to the type of *Echinacea* preparation [32].

In the current study, induction of AMI resulted in a progressive reduction of serum GM-CSF reaching the lowest level on day 14 of MI. Such a reduction in GM-CSF might be attributed to its uptake by polymorphonuclear cells (PMNs). In response to MI injury, mature PMNs in the BM are recruited to the myocardium where their chemoattraction is regulated by several chemokines and cytokines [33, 34]. These PMNs are able to clear up GM-CSF from the circulation [35]. Also, an increased circulating level of GM-CSF is a basal part of the persistent inflammatory reaction during cardiac repair which may augment cardiac dysfunction through the increased oxidative stress and the enhanced cardiomyocyte apoptosis and extracellular matrix degradation [36]. Therefore, continued reduction of serum GM-CSF levels by treatment with Avemar or by post-treatment with *Echinacea* is assumed to be beneficial in preventing left ventricular remodeling. Moreover, the lowered serum GM-CSF by Avemar treatment or by post-treatment with *Echinacea* is assumed to provide a chance for more CD34^+^ cells to differentiate into cardiomyocytes or endothelial cells rather than into granulocytes or macrophages. On the other hand, in groups pre- and post-treated with *Echinacea*, the GM-CSF level was increased on day 14 which might be attributed to the immune enhancing properties of *Echinacea*. GM-CSF transcription was reported to be downregulated by the rhinovirus and upregulated to some extent by treatment with *Echinacea* which supports this assumption [37]. These differences in serum GM-CSF levels between *Echinacea*-treated groups may account for the histopathological findings where the group post-treated with *Echinacea* showed no inflammatory reaction while the pre- and post-treated group showed a mild inflammatory reaction.

Immunohistochemical data in the current study revealed successful recruitment and homing of the mobilized CD34^+^ cells to the damaged myocardium in the case of post- treatment with *Echinacea* and pre- and post-treatment with Avemar or *Echinacea*. The claimed participation of these recruited CD34^+^ cells in myocardial regeneration after AMI in response to the above treatment may involve either direct incorporation of these cells into the newly developing vasculature and/or the production and secretion of angiogenic cytokines [38, 39]. Parallel to this finding, α-SMA-positive cells were increased in Avemar-treated groups with both vascular and separate scattered myofibroblast-like cells. Likewise, in *Echinacea*-treated groups, α-SMA-positive cells mostly gained histological features characteristic of pericytes in the wall of newly formed capillaries. In addition, a predominant presence of CD34^+^ cells in the endothelium of several vessels suggested their transdifferentiation into endothelial cells which may contribute to the significantly higher capillary density in the treated groups, particularly in *Echinacea* groups. Therefore, the overall increase in capillary density and transdifferentiation into vascular smooth muscle cells seen in the treated groups may ultimately help in regeneration of damaged myocardium. It was reported that endothelial progenitor cells derived from CD34^+^ cells could differentiate to endothelial- and smooth muscle-like cells and contribute to the development of cooperative vascular networks [40]. The possible mechanism of Avemar and *Echinacea* in recruitment and homing of SCs may depend on their antioxidant properties. The extracts of all *Echinacea* species and likewise Avemar contain several polyphenolic compounds and large amounts of flavonoids, having a potent antioxidant activity [41, 42]. Antioxidants have also been shown to play a major role in the regenerative potential of muscle-derived SCs in skeletal and cardiac muscles [43], as well as in enhancing the proliferation of adipose-derived mesenchymal SCs in vitro [44]. Other unknown mechanisms are not ruled out and need further research.

Histopathological changes in the heart from AMI animals might be attributed partially to the state of oxidative stress following isoprenaline injection which directly harms the cardiomyocytes and endothelial cells [45]. Treatment with Avemar or *Echinacea* resulted in reduced inflammatory reactions in the myocardial tissues which could be explained in the case of Avemar by its anti-inflammatory effect through the inhibition of the cyclooxygenases [46], while in *Echinacea* it could be attributed to the inhibition of free radical production in the development of inflammation [47]. Such a reduction in inflammatory reactions by Avemar and/or the inhibition of free radical production by *Echinacea* may help in creating a supportive environment for the homing SCs at the myocardium to promote their survival and ultimately may enhance their regenerative potentials.

## Conclusion

In conclusion, for the first time, we showed that administration of Avemar and *Echinacea* extracts effectively triggered the mobilization and homing of CD34^+^ stem cells to the infarcted myocardium and thus may enhance SC-based regeneration of the myocardial tissue. The potential of these safe and cheap products as additional therapeutic agents holds great promise for MI. Mechanisms of SC mobilization by these products needs to be unraveled.

## Abbreviations

AMI: Acute myocardial infarction
BM: Bone marrow
CK: Creatine kinase
ELISA: Enzyme-linked immunosorbent assay
G-CSF: Granulocyte colony-stimulating factor
GM-CSF: Granulocyte macrophage colony-stimulating factor
H&E: Hematoxylin and eosin
IL: Interleukin
MI: Myocardial infarction
PMN: Polymorphonuclear cell
SC: Stem cell
SDF-1: Stromal cell-derived factor-1
VEGF: Vascular endothelial growth factor
α-SMA: Alpha-smooth muscle actin

## References

[1] Chamuleau SAJ, Vrijsen KR, Rokosh DG, Tang XL, Piek JJ, Bolli R (2009). Cell therapy for ischaemic heart disease: focus on the role of resident cardiac stem cells. Neth Hear J..

[2] Chen F-M, Wu L-A, Zhang M, Zhang R, Sun H-H (2011). Homing of endogenous stem/progenitor cells for in situ tissue regeneration: promises, strategies, and translational perspectives. Biomaterials..

[3] Kränkel N, Spinetti G, Amadesi S, Madeddu P (2011). Targeting stem cell niches and trafficking for cardiovascular therapy. Pharmacol Ther..

[4] Vandervelde S, Van Luyn MJA, Tio RA, Harmsen MC (2005). Signaling factors in stem cell-mediated repair of infarcted myocardium. J Mol Cell Cardiol..

[5] Sanganalmath SK, Abdel-Latif A, Bolli R, Xuan Y, Dawn B (2011). Hematopoietic cytokines for cardiac repair: mobilization of bone marrow cells and beyond. Basic Res Cardiol..

[6] Telekes A, Hegedus M, Chae CH, Vékey K (2009). Avemar (wheat germ extract) in cancer prevention and treatment. Nutr Cancer..

[7] Iyer A, Brown L (2011). Fermented wheat germ extract (Avemar) in the treatment of cardiac remodeling and metabolic symptoms in rats. Evid Based Complement Altern Med..

[8] Wang C-Y, Chiao M-T, Yen P-J, Huang W-C, Hou C-C, Chien S-C (2006). Modulatory effects of *Echinacea* purpurea extracts on human dendritic cells: a cell- and gene-based study. Genomics..

[9] Kapai N, Anisimova NY, Kiselevskii MV, Sitdikova SM, Slavetskaya MB (2011). Selective cytokine-inducing effects of low dose *Echinacea*. Bull ExpBiol Med..

[10] Baean BJ, Ewiski FR, Zdanowski R (2012). Immunotropic activity of *Echinacea*. Part I. History and chemical structure. Centr Eur J Immunol.

[11] Rona G, Kahn DS, Chappel CI (1963). Studies on infarct-like myocardial necrosis produced by isoproterenol: a review. Rev Can Biol..

[12] Heimbach JT, Sebestyen G, Semjen G, Kennepohl E (2007). Safety studies regarding a standardized extract of fermented wheat germ. Int J Toxicol..

[13] Zhai Z, Haney D, Wu L, Solco A, Murphy PA, Wurtele ES (2007). Alcohol extracts of *Echinacea* inhibit production of nitric oxide and tumor necrosis factor-alpha by macrophages in vitro. Food AgricImmunol..

[14] Acikel M, Buyukokuroglu ME, Erdogan F, Aksoy H, Bozkurt E, Senocak H (2005). Protective effects of dantrolene against myocardial injury induced by isoproterenol in rats: biochemical and histological findings. Int J Cardiol..

[15] Tomita S, Li RK, Weisel RD, Mickle DA, Kim EJ, Sakai T (1999). Autologous transplantation of bone marrow cells improves damaged heart function. Circulation..

[16] Tsutsumi Y, Serizawa A, Kawai K (1995). Enhanced polymer one-step staining (EPOS) for proliferating cell nuclear antigen and Ki-67 antigen-applications to intraoperative frozen diagnosis. Pathol Int..

[17] Zhao Q, Sun C, Xu X, Zhou J, Wu Y, Tian Y (2011). CD34+ cell mobilization and upregulation of myocardial cytokines in a rabbit model of myocardial ischemia. Int J Cardiol..

[18] Leone AM, Rutella S, Bonanno G, Abbate A, Rebuzzi AG, Giovannini S (2005). Mobilization of bone marrow-derived stem cells after myocardial infarction and left ventricular function. Eur Hear J..

[19] Lapidot T, Petit I (2002). Current understanding of stem cell mobilization: the roles of chemokines, proteolytic enzymes, adhesion molecules, cytokines, and stromal cells. Exp Hematol..

[20] Fibbe WE, Pruijt JF, Van Kooyk Y, Figdor CG, Opdenakker G, Willemze R (2000). The role of metalloproteinases and adhesion molecules in interleukin-8-induced stem-cell mobilization. Semin Hematol..

[21] Thippeswamy BS, Thakker SP, Tubachi S, Kalyani GA, Netra MK, Patil U (2009). Cardioprotective effect of cucumistrigonusroxb on isoproterenol-induced myocardial infarction in rat. Am J Pharmacol Toxicol..

[22] Rathore N, John S, Kale M, Bhatnagar D (1998). Lipid peroxidation and antioxidant enzymes in isoproterenol induced oxidative stress in rat tissues. Pharmacol Res..

[23] Chung S, Dzeja PP, Faustino RS, Perez-Terzic C, Behfar A, Terzic A (2007). Mitochondrial oxidative metabolism is required for the cardiac differentiation of stem cells. Nat Clin Pr Cardiovasc Med..

[24] Chung S, Dzeja PP, Faustino RS, Terzic A (2008). Developmental restructuring of the creatine kinase system integrates mitochondrial energetics with stem cell cardiogenesis. Ann N Y Acad Sci..

[25] Kamihata H, Matsubara H, Nishiue T, Fujiyama S, Tsutsumi Y, Ozono R (2001). Implantation of bone marrow mononuclear cells into ischemic myocardium enhances collateral perfusion and regional function via side supply of angioblasts, angiogenic ligands, and cytokines. Circulation..

[26] Takahashi K, Ito Y, Morikawa M, Kobune M, Huang J, Tsukamoto M (2003). Adenoviral-delivered angiopoietin-1 reduces the infarction and attenuates the progression of cardiac dysfunction in the rat model of acute myocardial infarction. Molther..

[27] Kodama Y, Kitta Y, Nakamura T, Takano H (2006). Atorvastatin increases plasma soluble Fms-like tyrosine kinase-1 and decreases vascular endothelial growth factor and placental growth factor in association with improvement of ventricular function in acute myocardial infarction. J Am Coll Cardiol..

[28] Shintani S, Murohara T, Ikeda H, Ueno T, Honma T, Katoh A (2001). Mobilization of endothelial progenitor cells in patients with acute myocardial infarction. Circulation..

[29] Cangiano E, Cavazza C, Campo G, Valgimigli M, Francolini G, Malagutti P (2011). Different clinical models of CD34+ cells mobilization in patients with cardiovascular disease. J Thromb Thrombolysis..

[30] Schömig K, Busch G, Steppich B, Sepp D, Kaufmann J, Stein A (2006). Interleukin-8 is associated with circulating CD133+ progenitor cells in acute myocardial infarction. Eur Hear J..

[31] Roos D (2012). A single dose of granulocyte-macrophage colony-stimulating factor induces systemic interleukin-8 release and neutrophil activation in healthy volunteers. Blood..

[32] Ernesto AB, Haeri RS, Angela B. Method of augmenting the immune-modulatory activity of standardized *Echinacea* preparations. 2005: PAT-US20050175721. http://www.google.com/patents/CN1680584A?cl=en.

[33] Akpek M, Kaya MG, Lam YY, Sahin O, Elcik D, Celik T (2012). Relation of neutrophil/lymphocyte ratio to coronary flow to in-hospital major adverse cardiac events in patients with ST-elevated myocardial infarction undergoing primary coronary intervention. Am J Cardiol..

[34] Ma Y, Yabluchanskiy A, Lindsey ML (2013). Neutrophil roles in left ventricular remodeling following myocardial infarction. Fibrogenes Tissue Repair..

[35] Meisenberg B, Miller WMR (1998). Efficient and predictable mobilization of peripheral blood stem cells with low-dose cyclophosphamide followed by sequential GM-CSF and GCSF. Transfusion..

[36] Parissis JT, Adamopoulos S, Venetsanou K, Kostakis G, Rigas A, Karas SM (2004). Plasma profiles of circulating granulocyte-macrophage colony-stimulating factor and soluble cellular adhesion molecules in acute myocardial infarction. Contribution to post-infarction left ventricular dysfunction. Eur Cytokine Netw.

[37] Altamirano-Dimas M, Sharma M, Hudson JB (2009). *Echinacea* and anti-inflammatory cytokine responses: results of a gene and protein array analysis. Pharm Biol..

[38] Mackie AR, Losordo DW (2011). CD34-positive stem cells in the treatment of heart and vascular disease in human beings. Tex Hear Inst J..

[39] Iwasaki H, Kawamoto A, Ishikawa M, Oyamada A, Nakamori S, Nishimura H (2006). Dose-dependent contribution of CD34-positive cell transplantation to concurrent vasculogenesis and cardiomyogenesis for functional regenerative recovery after myocardial infarction. Circulation..

[40] Guo S, Cheng Y, Ma Y, Yang X (2010). Endothelial progenitor cells derived from CD34+ cells form cooperative vascular networks. Cell Physiol Biochem..

[41] Sloley BD, Urichuk LJ, Tywin C, Coutts RT, Pang PK, Shan JJ (2001). Comparison of chemical components and antioxidants capacity of different *Echinacea* species. J Pharm Pharmacol..

[42] Ehrenfeld M, Blank M, Shoenfeld Y, Hidvegi M (2001). AVEMAR (a new benzoquinone-containing natural product) administration interferes with the Th2 response in experimental SLE and promotes amelioration of the disease. Lupus..

[43] Urish KL, Vella JB, Okada M, Deasy BM, Tobita K, Keller BB (2009). Antioxidant levels represent a major determinant in the regenerative capacity of muscle stem cells. Mol Biol Cell..

[44] Sun L-Y, Pang C-Y, Li D-K, Liao C-H, Huang W-C, Wu C-C (2013). Antioxidants cause rapid expansion of human adipose-derived mesenchymal stem cells via CDK and CDK inhibitor regulation. J Biomed Sci..

[45] Upaganlawar AGH, Balaraman R (2011). Isoproterenol induced myocardial infarction: protective role of natural products. J Pharmacol Toxicol..

[46] Boros LG, Nichelatti M, Shoenfeld Y (2005). Fermented wheat germ extract (Avemar) in the treatment of cancer and autoimmune diseases. Ann NY Acad Sci..

[47] Speroni E, Govoni P, Guizzardi S, Renzulli C, Guerra MC (2002). Anti-inflammatory and cicatrizing activity of *Echinacea* pallida Nutt root extract. J Ethnopharmacol..

